# Abundance of Class 1 Integron-Integrase and Sulfonamide Resistance Genes in River Water and Sediment Is Affected by Anthropogenic Pressure and Environmental Factors

**DOI:** 10.1007/s00248-016-0843-4

**Published:** 2016-09-06

**Authors:** Ryszard Koczura, Joanna Mokracka, Agata Taraszewska, Natalia Łopacinska

**Affiliations:** Department of Microbiology, Faculty of Biology, Adam Mickiewicz University in Poznań, ul. Umultowska 89, 61-614 Poznań, Poland

**Keywords:** Integron, Wastewater, Surface water, Seasonality

## Abstract

In this study, we determined the presence of class 1 integron-integrase gene in culturable heterotrophic bacteria isolated from river water and sediment sampled upstream and downstream of a wastewater treatment plant effluent discharge. Moreover, we quantified *intI1* and sulfonamide resistance genes (*sul1* and *sul2*) in the water and sediment using qPCR. There was no correlation between the results from water and sediment samples, which suggests integron-containing bacteria are differentially retained in these two environmental compartments. The discharge of treated wastewater significantly increased the frequency of *intI1* among culturable bacteria and the gene copy number in river water, and increased the number of *sul1* genes in the sediment. We also observed seasonal differences in the frequency of the class 1 integron-integrase gene among culturable heterotrophs as well as *intI1* copy number in water, but not in sediment. The results suggest that the abundance of class 1 integrons in aquatic habitat depends on anthropogenic pressure and environmental factors.

**Electronic supplementary material:**

The online version of this article (doi:10.1007/s00248-016-0843-4) contains supplementary material, which is available to authorized users.

## Introduction

Surface waters act as important recipients, reservoirs, and vectors of biotic contaminants like antibiotic-resistant bacteria (ARBs) and antibiotic resistance genes (ARGs) in the environment [1–3]. Antibiotic-resistant bacteria can enter the environment through, for example, wastewater treatment plant effluents, and agricultural runoff. Agricultural and wastewater input of antibiotics, biocides, and heavy metals into surface waters imposes selective pressure enabling the maintenance and amplification of ARBs and enhancing lateral transfer of ARGs in the environment [4, 5].

Class 1 integrons are genetic assembly elements involved in capture and spread of ARGs. They are characterized by a 5′-conserved region that consists of an integrase gene *intI*, attachment site *attC*, and a promoter P_C_ that directs transcription of the incorporated genes. The 3′-conserved region contains *qacΔE1* and *sul1* genes responsible for resistance to quaternary ammonium compounds and sulfonamides, respectively [6, 7]. Class 1 integrons capture gene cassettes that determine resistance to antimicrobials, code for transport proteins, esterases, phosphatases, transposases, and also proteins of unknown function. Over 130 different resistance gene cassettes have been described in the variable regions of class 1 integrons, so far. These integrons are widely distributed among clinical strains, and in environmental isolates, their presence can be associated with increased frequency of virulence genes [8]. Class 1 resistance integrons are located on mobile elements like transposons and plasmids and thus are involved in spread of antibiotic resistance genes in bacteria by lateral gene transfer [9, 10].

Integrons have been detected in the genomes of cultivable heterotrophs isolated from surface waters with different anthropogenic pressure, wastewater, and ground waters [3, 11, 12]. However, culture-based methods do not take into account nonculturable microorganisms which constitute the vast majority of environmental microorganisms [13]. The prevalence of integrons is proposed to serve as a marker of antibiotic resistance level [14] and anthropogenic pollution in the environment [15]. Investigations into the levels of *intI1* and *sul* genes along a gradient of anthropogenic impact not only can give insights into the spread and proliferation of ARGs in surface water but also can identify critical point sources of ARGs in river water [15].

The aim of this study was to evaluate the copy number and abundance of class 1 integron-integrase and sulfonamide resistance genes in river water and sediment at three sites with different anthropogenic pressure. We tested hypotheses that the copy number and/or relative abundance of class 1 integron-integrase and sulfonamide resistance genes in river habitat (a) are affected by anthropogenic pressure and (b) show seasonal fluctuations. Additionally, we set up another hypothesis that water and sediment microbiomes may differ in the abundance of the aforementioned genes.

## Methods

### Sample Collection

Water and sediment samples were taken monthly from January to December 2014 from the Warta River. Altogether, there were 12 sampling events: 3 for each season—January, February, and March in winter; April, May, and June in spring; July, August, and September in summer; and October, November, and December in autumn. The Warta River is 808-km long and flows in central-western Poland. There were three sampling sites localized ca. 15 km away from each other: site no. 1 (referred to as “upstream”) in Rogalinek (GPS coordinates 52.2488; 16.8948), under relatively low anthropogenic pressure, localized upstream of a municipal water intake; site no. 2 (referred to as “city”) in Poznań (GPS 52.3814; 16.9362), a city with a population of 550,000 and several hospitals without their own wastewater treatment facilities; and site no. 3 (referred to as “downstream”) near Czerwonak (GPS 52.4946; 16.9734), 5 km downstream of the outlet of a municipal wastewater treatment plant serving Poznań and the nearby areas. The wastewater treatment plant (WWTP) is capable of taking up 200,000 m^3^ of sewage per day. During sewage treatment, the chemical oxygen demand is reduced from 1158 to 36 mg O_2_ L^−1^, biological oxygen demand from 483 to 3.5 mg O_2_ L^−1^, total suspended solids from 555 to 5 mg L^−1^, total nitrogen from 88 to 10 mg N L^−1^, and total phosphorus from 15 to 0.6 mg P L^−1^. The water samples were collected to sterile containers from the riverbed. The sediment samples were taken 10 cm below the bottom surface. The samples were transported to laboratory at 4 °C and processed within 3 h.

Physicochemical parameters of water, namely temperature, pH, total dissolved solids (TDS), salinity, conductivity, and rugged dissolved oxygen (RDO) concentration, were measured in situ with the use of a portable multiparameter meter OrionStar A329 (Thermo Scientific). Biological oxygen demand (BOD) was measured with OxiTop Control system (WTW).

### Bacterial Cultures

Total number of culturable heterotrophic bacteria was determined by pour-plate method on Brain Heart Infusion agar (bioMérieux). Series of logarithmic dilutions (10^−1^ to 10^−4^) of water and sediment samples were prepared in sterile saline, added to the medium and the plates incubated at 25 °C for 72 h. Coliform bacteria were counted on Brilliance E. coli/Coliform Selective Agar (Oxoid). Serial dilutions (up to 1:1000) were inoculated on the surface of the medium and the plates were incubated at 37 °C for 24 h.

### DNA Template Preparation

For DNA templates from bacterial isolates, bacterial colonies were suspended in sterile H_2_O and lysed by heating (95 °C for 2 min). The lysates were stored at −20 °C [16]. Clonal relatedness of the isolates was determined by the ERIC-PCR method according to Versalovic et al. [17], followed by band patterns analysis with the use of GelCompar II 3.5 software (Applied Maths) with Dice similarity coefficient and unweighted pair-group method with average linkages (UPGMA) clustering method. Only genetically unrelated isolates with DNA fingerprinting pattern similarity below 90 % were further analyzed.

For isolation of total DNA from water metagenome, 3 L of water samples were filtered through 8- and 0.45-μm cellulose nitrate filters (Sartorius Stedim). Material deposited on filters was washed off with water, and then total DNA was extracted by heat lysis and with the use of Genomic Mini kit (A&A Biotechnology) [18]. Total DNA from sediment metagenome was isolated by heat lysis and Genomic Mini kit. The DNA samples were further purified with Anty-Inhibitor kit (A&A Biotechnology) to remove PCR inhibitors. The quality of DNA was assessed spectrophotometrically (A260/280 ratio 1.7–2.0) and by agarose gel electrophoresis.

### Detection of Integron-Integrase Genes by Conventional PCR

The frequency of class 1, 2, and 3 integrons among culturable heterotrophs was determined by multiplex PCR assay [16] for the detection of *intI1*, *intI2*, and *intI3* integron-integrase genes. For the assay, 96 nonrepetitive isolates cultured on Brain Heart Infusion agar were randomly chosen from each sampling site at each sampling event (6912 isolates in total). PCR amplifications were carried out in a C1000 Touch thermal cycler (Bio-Rad), in a 15-μL volume with 1 μL of genomic DNA, PCR buffer, 0.25 μM of each primer (Oligo.pl), 200 μM of dNTP mix (Novazym), 2.5 mM of MgCl_2_, and 0.5 U of DreamTaq DNA polymerase (Thermo Scientific). The amplicons were separated in 2 % Nova Mini agarose gel (Novazym). The molecular size of the PCR products was determined with GelCompar II 3.5 (Applied Maths). Positive and negative controls were included in each reaction. The authenticity of amplicons was confirmed by sequencing.

### Quantification of Integron-Integrase and *sul* Genes by qPCR

Quantitative real-time PCR (qPCR) was used for determination of the abundance of integron-integrase and sulfonamide resistance genes in total DNA samples. We quantified genes detected with high frequency in the genomes of culturable heterotrophic bacteria: *intI1*, *sul1*, and *sul2* (data for *sul1* and *sul2* not shown). The sequences of primers targeting *intI1* have been recommended by Barraud et al. [19], whereas those complementary to *sul1* and *sul2* genes have been designed by Pei et al. [20] (Supplementary Table S1). The gene quantities were normalized to 16S ribosomal RNA (rRNA) gene copy number, and the relative abundance values were expressed as percentages and calculated using the formula: [(*intI*/16S) × 4 × 100], with four being the average number of copies of the gene encoding 16S rRNA per bacterial cell, according to the ribosomal RNA database [3, 21].

Standard curves for qPCR were constructed from serial dilutions of purified PCR products ranging from 10^1^ to 10^8^ gene copies per microliter. Reactions were carried out in 96-well plates in a final volume of 20 mL with Luminaris Color HiGreen qPCR Master Mix (Thermo Scientific). Specificity of amplification was determined by melt curve analysis and gel electrophoresis. Reactions were carried out in a CFX96 Touch Real-Time PCR Detection System and analyzed with CFX Manager 3.1 software (Bio-Rad).

### Statistical Analysis

Physicochemical parameters of water were compared by using Mann–Whitney *U* and Kruskal–Wallis tests. Differences in the frequency of integron-integrase genes among culturable bacteria were determined by Fisher’s exact test. Differences in the number of bacteria as well as gene copy numbers and abundance between the sampling sites were assessed pairwise with Mann–Whitney *U* test. Differences in gene copy numbers in different sampling seasons were assessed with Kruskal–Wallis test. Correlations between physicochemical parameters and gene copy numbers were determined with Spearman’s rank correlation coefficient. Calculations were done with Statistica 12 software (StatSoft). *P* < 0.05 was used to reflect statistical significance.

## Results

### Physicochemical Parameters of Water

The characteristics of Warta River water at the three sampling sites are presented in Supplementary Table S2. There were no significant differences between the values recorded at different sites with the exception of salinity, which at the downstream site was higher when compared with the upstream site (*P* = 0.024). Seasonal differences (*P* < 0.001), however, were noticed between temperature, pH, conductivity, TDS concentration, salinity, and BOD measured in winter (January–March), spring (April–June), summer (July–September), and autumn months (October–December) (Supplementary Table S3).

### Culturable Heterotrophic Bacteria

The highest average total number of heterotrophic bacteria in water samples was noted downstream from the WWTP—2.0 × 10^4^ CFU mL^−1^ (Supplementary Table S4). It was significantly higher compared with 2.3 × 10^3^ CFU mL^−1^ at the upstream site (*P* = 0.006) and 3.9 × 10^3^ CFU mL^−1^ at the city site (*P* = 0.023). The highest average number of heterotrophic bacteria in the sediment was also noted at the downstream site, where it reached 1.1 × 10^6^ CFU g^−1^, compared with 1.9 × 10^5^ CFU g^−1^ at the upstream site and 5.0 × 10^5^ CFU g^−1^ at the city site.

The average number of coliform bacteria in the water was the highest downstream from the WWTP (5.4 × 10^2^ CFU mL^−1^) (Table 1); however, it did not differ significantly from those noted for the upstream site (2.0 × 10^2^ CFU mL^−1^) and the city site (3.4 × 10^2^ CFU mL^−1^). The number of coliforms in the sediment was highest downstream from the WWTP (4.9 × 10^4^ CFU g^−1^), significantly higher than at the upstream site (8.6 × 10^3^ CFU g^−1^, *P* = 0.004) and the city site (1.0 × 10^4^ CFU g^−1^, *P* = 0.009).

**Table 1:** Correlation between physicochemical parameters of water and bacterial counts, *intI1* frequency among culturable bacteria, and copy number and abundance of 16S rRNA gene, *intI1*, *sul1*, and *sul2* genes in water

	Culturable heterotrophs	Coliforms	*intI1* frequency	16S copy number	*intI1* copy	*intI1* abundance	*sul1* copy number	*sul1* abundance	*sul2* copy number	*sul2* abundance
Temperature	−0.067	*0.580*	−*0.439*	0.300	−0.192	−0.367	0.158	−0.110	0.156	−0.077
pH	−0.009	0.326	−*0.484*	0.323	−0.122	−0.302	*0.366*	0.063	0.220	0.021
Conductivity	0.019	−*0.376*	*0.496*	−*0.380*	*0.398*	*0.596*	0.140	*0.376*	0.080	*0.428*
TDS	−0.061	−*0.330*	*0.479*	−*0.414*	*0.357*	*0.595*	0.174	*0.415*	0.133	*0.491*
Salinity	0.053	−0.260	*0.442*	−0.257	*0.416*	*0.511*	0.124	0.281	0.128	*0.457*
RDO	−0.078	−0.314	0.173	−*0.482*	0.159	*0.499*	*0.457*	*0.669*	0.136	0.296
BOD	−0.226	0.235	−0.025	0.205	−*0.374*	−*0.479*	0.167	−0.034	*0.441*	0.231

Spearman’s rank correlation coefficient value. Statistically significant correlations (*P* < 0.05) are given in italics

### Integron-Integrase Genes in Culturable Bacteria

Class 1 integron-integrase genes were detected in the genomes of culturable heterotrophs in water and sediment samples from all sites. Single isolates with class 2 integron-integrase genes were detected once in water sampled at the city site and once in water sampled downstream from the WWTP, whereas in sediment samples they were not found. Class 3 integron-integrase gene was not detected.

In water samples, the highest average frequency of class 1 integron-integrase gene in heterotrophic isolates was found in those originating from the downstream site—7.7 % (Supplementary Table S4), whereas the frequency in upstream and city sites was 3.8 and 3.6 %, respectively (*P* < 0.001). A similar trend was observed in the case of bacteria cultured from sediment; the highest frequency of *intI1* gene was noticed among isolates from the downstream site (3.9 %), whereas at the upstream and city sites, the *intI1* frequency was significantly lower: 1.6 and 1.3 %, respectively (*P* < 0.001).

The frequency of *intI1* gene in culturable bacteria isolated from each sampling site was significantly higher in water compared with sediment (*P* ≤ 0.001); however, there was no correlation between the values for water and sediment isolates.

We observed seasonal differences in the frequency of class 1 integron-integrase gene in culturable bacteria in water (Supplementary Table S5). The highest was observed in winter—9.9 %, followed by spring—5.0 %, autumn—2.9 %, and summer months—0.9 % (*P* = 0.005). On the other hand, in the sediment samples, higher frequency of *intI1* gene occurred in spring and autumn (3.5 and 3.6 %, respectively) than in other seasons, but the differences were not significant (*P* = 0.132).

When it comes to physicochemical parameters, the frequency of *intI1* gene in water isolates was negatively correlated with temperature and pH of water, and positively correlated with conductivity, salinity, and concentration of total dissolved solids (Table 1). The frequency of class 1 integron-integrase gene in bacteria cultured from sediment samples was not correlated with any of the parameters.

### Class 1 Integron-Integrase Gene in Total DNA

We quantified *intI1* integrase gene and sulfonamide resistance genes *sul1* and *sul2* as well as 16S rRNA gene in total DNA samples of water and sediment metagenomes. The average numbers of 16S rRNA gene copies in water samples were similar at the three sampling sites. The average numbers of 16S rRNA gene copies in sediment samples at the three sites were similar as well (Supplementary Table S4). The average number of 16S rRNA gene copies per gram of the sediment samples was two orders of magnitude higher than the number of 16S rRNA gene copies per milliliter of the water.

**Fig 1:**
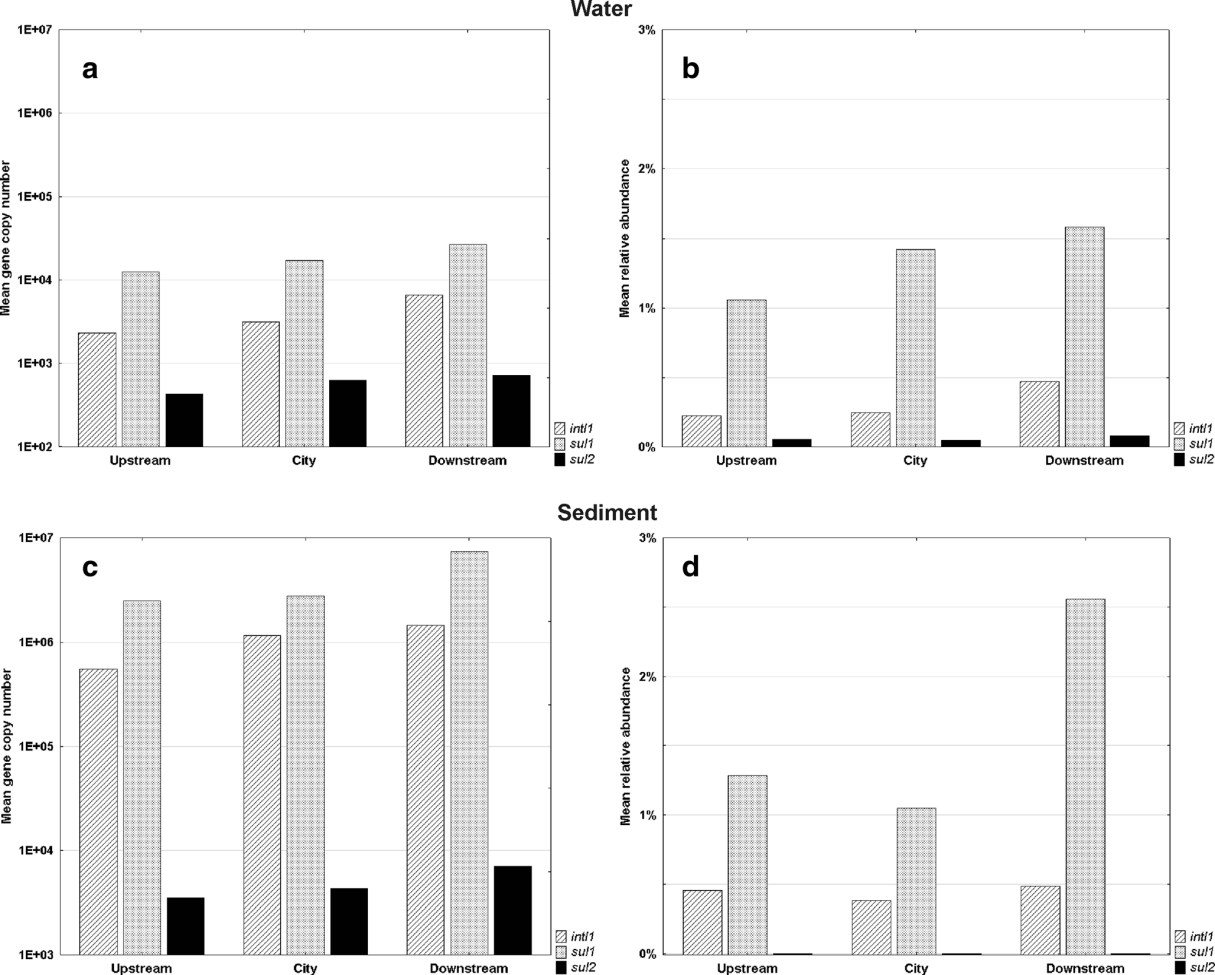
Mean copy number and abundance of *intI1*, *sul1*, and *sul2* genes in river water (**a**, **b**) and sediment (**c**, **d**) sampled at upstream, city, and downstream sites

The average *intI1* gene copy number in water samples was the highest downstream from the WWTP (6.6 × 10^3^ copies mL^−1^) and differed significantly from the upstream site (2.3 × 10^3^ copies mL^−1^, *P* = 0.006) and the city site (3.1 × 10^3^ copies mL^−1^, *P* = 0.030) (Fig. 1, Supplementary Table S4). Relative abundance of class 1 integron-integrase gene ranged from 0.22 to 0.47 %, but the differences were not statistically significant. No significant differences between the three sites were found for the sediment samples; the gene copy number ranged from 5.5 × 10^5^ to 1.4 × 10^6^ copies g^−1^, whereas the abundance ranged from 0.38 to 0.49 %.

There was no correlation between the *intI1* copy number or abundance and the frequency of the gene in culturable heterotrophic bacteria.

The copy number of the integrase gene in the sediment was significantly higher than in the water. However, since there was a higher number of 16S rRNA genes in the sediment, the relative abundance of *intI1* gene in the two sample types was similar. There was no correlation between the copy number and abundance of the *intI1* gene in water and sediment.

There were seasonal differences in the copy number (*P* = 0.017) and abundance (*P* = 0.011) of *intI1* gene in water. Higher copy number of class 1 integron-integrase gene was noticed in winter and summer (5.0 × 10^3^ and 5.0 × 10^3^ copies mL^−1^, respectively). The highest relative abundance of *intI1* gene was recorded during winter months (0.65 %). When it comes to seasonal changes in the river sediment, there were no significant differences between the seasons regarding both the copy number and the abundance of class 1 integron-integrase gene (Supplementary Table S5, Supplementary Fig. S1).

Regarding physicochemical parameters, the *intI1* copy number and abundance showed moderate positive correlation with water conductivity, TDS, and salinity, and negative correlation with biological oxygen demand. The *intI1* abundance was also positively correlated with the concentration of dissolved oxygen (Table 1).

### Sulfonamide Resistance Genes in Total DNA Samples

The copy number of *sul1* gene in water samples ranged from 1.2 × 10^4^ to 2.7 × 10^4^ copies mL^−1^, but the differences between the three sites were not significant. The *sul2* gene was present in much lower quantities (Fig. 1, Supplementary Table S4). The relative abundance of *sul1* was similar at the three sites and ranged from 1.06 to 1.56 %. The *sul2* gene abundance was lower, from 0.051 to 0.083 %. Differences between the abundances of *sul1* and *sul2* at the three sampling sites were not statistically significant.

In the sediment, the highest average copy number of *sul1* was noted downstream the wastewater treatment plant (7.4 × 10^6^ copies g^−1^) and the lowest at the upstream site—2.5 × 10^6^ copies g^−1^ (*P* = 0.040). On the other hand, the differences between *sul1* abundances at the three sampling sites were not statistically significant. The relative abundance of *sul1* ranged from 1.05 to 2.56 %, whereas *sul2* was less abundant (0.00006 to 0.00024 %).

The copy number of the *sul1* and *sul2* sulfonamide resistance genes in the sediment was significantly higher (*P* < 0.001 and *P* = 0.043, respectively) than in the water. The relative abundance of *sul1* was in water and sediment was similar; however, *sul2* was significantly more abundant in the water samples (*P* < 0.001). There was no correlation between the copy number and abundance of particular genes in water and sediment.

No statistically significant seasonal changes were found in the copy number and abundance of *sul1* gene in river water and sediment. On the other hand, *sul2* showed significantly higher abundance in water samples in the spring (*P* < 0.001) (Supplementary Fig. S1, Supplementary Table S5).

The number of *sul1* gene copies in water showed weak positive correlation with pH of water and moderate positive correlation with dissolved oxygen concentration (Table 1). The abundance of *sul1* was positively correlated with conductivity, TDS, and dissolved oxygen. The *sul2* copy number displayed moderate positive correlation BOD value, whereas relative abundance of this gene was positively correlated with conductivity, TDS, and salinity.

## Discussion

In this study, we characterized integron-integrase genes in the genomes of culturable bacteria and determined the copy number and relative abundance of class 1 integrons and sulfonamide resistance genes in river water and sediment at three sites with diverse anthropogenic impact. Moreover, we determined the copy number of 16S rRNA gene, which in the sediment was approximately two orders of magnitude higher than that in water. This also reflected the differences in the total number of culturable heterotrophic bacteria and coliforms between water and sediments. Higher number of 16S rRNA gene copies has been also reported by Chen et al. [22], who found approximately 1000 times higher number of 16S rRNA gene copies in the sediment of Pear River, China, compared with the water.

We have previously shown that discharge of effluent from the wastewater treatment plant located between the city site and the downstream site contributes to increased frequency of class 1 integrons among *E. coli* strains and elevates their antimicrobial resistance [23]. In the current study, we found that the frequency of *intI1* gene carriage among total heterotrophic bacteria was also significantly higher downstream from the WWTP, which indicates contribution of WWTP effluent to increased frequency of class 1 integrons not only among *E. coli* but also among other culturable heterotrophic bacteria. This was also confirmed by culture-independent approach, which showed approximately twofold increase in both the copy number of *intI1* gene per milliliter of water and its relative abundance at the downstream site compared to the two other sites. The lack of significant differences between the upstream and city sites suggests that the major contributor to anthropogenic pollution was the WWTP. The impact of the WWTP effluent on the level of *intI1* gene copy number and abundance has been also shown by LaPara et al. [24], Berglund et al. [25], and Makowska et al. [18]. The copy number of class 1 integron-integrase gene in water was comparable to that found in another Polish river, Zimny Potok [18]; however, Chen et al. [22] have analyzed the concentration of *intI1* in Pearl River, China, and reported values ranging from 1 × 10^5^ to 2 × 10^6^, which are two to three orders of magnitude higher. The abundance of *intI1* gene can be used as a measure of anthropogenic pollution in the environment. The *intI1* gene, linked to genes conferring resistance to antibiotics, disinfectants, and heavy metals, is found in a wide variety of pathogenic and nonpathogenic bacteria, which are selected by antibiotics, heavy metals, synthetic organic compounds, and other environmental contaminants. Due to rapid generation time of host cells and horizontal gene transfer, the abundance of *intI1* can change rapidly, and this way, it reflects the changes in pollution level in the environment [15].

The *intI1* genes occur naturally in environmental samples and their sequence shows considerable diversity. In environmental samples, both clinical and environmental genes *intI1* are present and amplified unless generic primers specific for clinical *intI1* are used. As we used universal starters, the data of quantification of *intI1* can be blurred by the presence of environmental *intI1*. Yet, it is known that most nonclinical *intI1* variants originate from relatively unpolluted areas [26].

The presence of clinical integron-integrases of other classes was rare. We found class 2 integron integrase in isolates cultured from single samples of water. Their incidence was less frequent than in the previous research concerning Warta River water [23].

Anthropogenic point sources of pollution have been reported to increase the abundance of *sul1* in river water and sediments [25, 27]. In our study, the copy number and relative abundance of *sul1* and *sul2* genes in the water showed similar trend as in the case of *intI1*; however, the differences were not statistically significant. The *sul1* gene is typically located in the 3′-conserved segment of class 1 integrons [28]. The copy number of *sul1* was higher than the copy number of *intI1*. Similar observation has been reported by Chen et al. [22] and Makowska et al. [18]. This suggests that *sul1* was localized also outside the integron structure. The *sul1* gene not associated with a class 1 integron has been reported, among others, for *Stenotrophomonas maltophilia* [29] and *Salmonella* spp. [30]. Of the two sulfonamide resistance genes, *sul1* was more abundant, both in water and sediment, which is in agreement with results presented by Gao et al. [31]. On the other hand, Jiang et al. [32] and Makowska et al. [18] have reported similar concentrations of *sul1* and *sul2* in river water. We found seasonal differences in the frequency of class 1 integrons in culturable bacteria. Higher occurrence of *intI1*-positive isolates was noticed during colder months. We also noted higher relative abundance of the *intI1* gene (but not *sul1* or *sul2*) in total DNA samples in cold months. This study; however, encompassed only 1 year of sampling, which is a limitation. Similar phenomena concerning coliform bacteria in lake waters have been observed by Koczura et al. [33]. The frequency of integron carriage among culturable heterotrophs was negatively correlated with temperature. Low temperature can affect horizontal gene transfer of integrons and ARGs due to enhanced competence of bacterial cells and transformation associated with stress response mechanisms [34]. Moreover, there is higher consumption of antibiotics in winter, so river water could have been contaminated by sublethal concentrations of antimicrobials through the discharge of treated wastewater or uncontrolled waste disposal [33]. Besides, low temperature decreases the rate of degradation of some antimicrobials in water [35]. Higher quantities of class 1 integron-integrase and antibiotic resistance genes in winter have been also found in Pearl River and its estuary, China; however, this was likely caused by a significant increase in the river runoff during wet season in summer [22]. On the other hand, no relationship between temperature and the concentration of integron-integrase and *sul* genes has been recorded in the water taken from the Daliaohe River and Liaohe River estuaries [36].

Spearman’s rank correlation showed some relationships between water parameters and quantity or abundance of ARGs. Conductivity, salinity, and dissolved oxygen concentration were positively correlated with *intI1* frequency, copy number, and/or relative abundance, as well as the abundance of sulfonamide resistance genes. Similar results concerning *sul* genes have been reported by Makowska et al. [18].

The copy numbers of *intI1*, *sul1*, and *sul2* genes in the sediment samples were significantly higher than those in the water, which was caused by higher number of bacterial cells, reflected by the amount of 16S rRNA gene. The significantly higher copy number of *intI1* in sediment can result from the higher number of bacteria and longer persistence of ARGs in sediment, which may constitute a major ARG reservoir in river sediment. Extracellular and intracellular DNA is more stable in sediment than in water and additionally, plasmid DNA, which harbors ARGs, is more persistent than chromosomal DNA [37]. It is difficult to compare water and sediment as they form two distinct compartments which differ in the abundance of microorganisms and concentrations of DNA. We used 16S rRNA gene to estimate the relative abundance of genes, but it does not take into account extracellular DNA which is present in both: water and sediment. The use of a given amount of foreign DNA as an internal control would allow determining the purity quotient and extraction efficiency [36]. The frequency of class 1 integrons in culturable bacteria and the abundance of *sul2* gene were higher in the water, whereas the abundance of *intI1* and *sul1* genes in water and sediment did not differ significantly. There was no correlation between the copy number and abundance of particular genes in water and sediment samples. This may suggest that in a river, water and sediment microbiomes differ in the frequency of class 1 integron-integrase and sulfonamide resistance genes.

In conclusion, we showed that class 1 integrons predominated among clinical integrons in bacteria cultured from river water and sediment. The frequency of carriage of class 1 integron-integrase gene by culturable heterotrophic bacteria as well as the copy number and abundance of *intI1* and sulfonamide resistance genes in the metagenome of water and sediment is affected by discharge of effluent from WWTP, which indicates that *intI1* can be used as a measure of anthropogenic impact. The results also suggest that river water and sediment microbiomes differ in the of class 1 integron-integrase and sulfonamide resistance genes. Moreover, there was temporal variation in the abundance of *intI1* and *sul* genes.

## Electronic supplementary material

Below is the link to the electronic supplementary material.

ESM 1 (DOCX 577 kb)
