# Supplementary material for: Abundance of Class 1 Integron-Integrase and Sulfonamide Resistance Genes in River Water and Sediment Is Affected by Anthropogenic Pressure and Environmental Factors
Source: Microb Ecol. 2016 Sep 6;72(4):909–16. doi: 10.1007/s00248-016-0843-4 (PMC5080314; doi:10.1007/s00248-016-0843-4)

**Abundance of class 1 and 2 integrase and sulfonamide resistance genes in Warta river water and sediments is affected by anthropogenic pressure and environmental factors**

Ryszard Koczura, Joanna Mokracka, Agata Taraszewska, Natalia Łopacinska

Department of Microbiology, Faculty of Biology, Adam Mickiewicz University in Poznań, 61-614 Poznań, Poland

**Supplementary Table S1** Primers and conditions of qPCR reactions

| Targeted gene | Primer name | Sequence | Amplification conditions | Reference |
| --- | --- | --- | --- | --- |
| 16S rRNA | Bact1369F | CGGTGAATACGTTCYCGG | 95°C for 10 min; 40 cycles of 95°C for 15 sec and 56°C for 1 min | [38] |
|  | Prok1492R | GGWTACCTTGTTACGACT |  |  |
| *intI1* | intI1-F | GCCTTGATGTTACCCGAGAG | 95°C for 10 min; 40 cycles of 95°C for 15 sec and 60°C for 1 min | [19] |
|  | intI1-R | GATCGGTCGAATGCGTGT |  |  |
| *sul1* | sul1-F | CGCACCGGAAACATCGCTGCAC | 95°C for 10 min; 40 cycles of 95°C for 15 sec and 65°C for 1 min | [20] |
|  | sul1-R | TGAAGTTCCGCCGCAAGGCTCG |  |  |
| *sul2* | sul2-F | TCCGGTGGAGGCCGGTATCTGG | 95°C for 10 min; 40 cycles of 95°C for 15 sec and 57.7°C for 1 min | [20] |
|  | sul2-R | CGGGAATGCCATCTGCCTTGAG |  |  |

**Supplementary Table S2** Mean values of physicochemical parameters of the Wart river water at three sampling sites

| Parameter [unit] | Site 1 (Rogalinek) | Site 2 (Poznań) | Site 3 (Czerwonak ) | Mean |
| --- | --- | --- | --- | --- |
| Temperature [°C] | 14.0±6.6 | 14.0±6.5 | 13.9±6.5 | 14.0±6.3 |
| pH | 8.27±0.26 | 8.23±0.29 | 8.20±0.22 | 8.23±0.25 |
| Conductivity [µS cm^-1^] | 574±55 | 594±65 | 620±63 | 596±62 |
| TDS^a^ [ppm] | 280±29 | 290±33 | 305±30 | 291±32 |
| Salinity [ppt] | 0.320±0.023 | 0.331±0.026 | 0.345±0.026 | 0.331±0.027 |
| RDO^b^ [mg L^-1^] | 9.87±1.24 | 9.70±1.27 | 9.71±1.13 | 9.76±1.18 |
| BOD^c^ [mg L^-1^] | 7.8±2.3 | 9.8±4.2 | 11.0±5.1 | 9.5±4.3 |

^a^ TDS – total dissolved solids

^b^ RDO – rugged dissolved oxygen

^c^ BOD – biological oxygen demand

**Supplementary Table S3** Mean values of physicochemical parameters of the Wart river water in different seasons

|  | Winter | Spring | Summer | Autumn |
| --- | --- | --- | --- | --- |
| Temperature [°C] | 6.4±1.9 | 17.5±4.1 | 20.6±3.1 | 11.4±3.0 |
| pH | 8.05±0.09 | 8.19±0.24 | 8.54±0.23 | 8.15±0.09 |
| Conductivity [µS cm^-1^] | 681±21 | 590±43 | 562±45 | 551±28 |
| TDS^a^ [ppm] | 334±10 | 290±21 | 276±22 | 265±15 |
| Salinity [ppt] | 0.364±0.012 | 0.333±0.020 | 0.321±0.023 | 0.310±0.015 |
| RDO^b^ [mg L^-1^] | 10.3±0.6 | 9.08±1.22 | 9.69±1.55 | 9.92±1.01 |
| BOD^c^ [mg L^-1^] | 6.1±1.6 | 14.6±4.8 | 8.9±1.6 | 8.5±2.7 |

^a^ TDS – total dissolved solids

^b^ RDO – rugged dissolved oxygen

^c^ BOD – biological oxygen demand

**Supplementary Table S4** Mean values of bacterial counts, *intI1* gene frequency among culturable bacteria, and copy number and abundance of 16S rRNA gene, *intI1*, *sul1* and *sul2* genes in water and sediment samples at three sampling sites

|  | Upstream site  n = 12 | City Site  n = 12 | Downstream site  n = 12 | Mean  n = 36 |
| --- | --- | --- | --- | --- |
| **Water** | | | | |
| Total heterotrophic bacteria [CFU mL^-1^] | 2.3×10^3^±2.0×10^3 a^  min 2.3×10^2^  max 7.6×10^3^ | 3.9×10^3^±4.8×10^3^  min 1.0×10^3^  max 1.5×10^4^ | 2.0×10^4^±2.9×10^4^  min 1.7×10^3^  max 8.4×10^4^ | 8.9×10^3^ ±1.8×10^4^  min 2.3×10^2^  max 8.4×10^4^ |
| Coliforms [CFU mL^-1^] | 2.0×10^2^±1.76×10^2^ min 4.0×10^1^  max 6.0×10^2^ | 3.4×10^2^±4.9×10^2^ min 3.6×10^1^  max 1.8×10^3^ | 5.4×10^2^×9.1×10^2^ min 9.5×10^1^  max 3.4×10^3^ | 3.6×10^2^±6.0×10^2^  min 3.6×10^1^  max 3.4×10^3^ |
| *intI1* frequency^b^ [%] | 3.8±5.6  min 0  max 19.8% | 3.6±3.6  min 0  max 9.4% | 7.7±8.4  min 1.0  max 27.1% | 5.0±6.3  min 0  max 27.1% |
| 16S rRNA gene [copies mL^-1^] | 8.8×10^6^±7.7×10^6^  min 1.3×10^6^  max 3.3×10^7^ | 9.1×10^6^±7.3×10^6^  min 2.0×10^6^  max 3.2×10^7^ | 1.1×10^7^±8.2×10^6^  min 1.8×10^6^  max 2.7×10^7^ | 9.7×10^6^ ±8.3×10^6^  min 1.3×10^6^  max 3.3×10^7^ |
| *intI1* [copies mL^-1^] | 2.3×10^3^±1.3×10^3^  min 6.5×10^2^  max 8.9×10^3^ | 3.1×10^3^±1.6×10^3^  min 7.7×10^2^  max 6.4×10^3^ | 6.6×10^3^±1.3×10^3^  min 1.1×10^3^  max 1.4×10^4^ | 4.0×10^3^±3.3×10^3^  min 6.5×10^2^  max 1.4×10^4^ |
| *intI1* abundance^c^ [%] | 0.22±0.24  min 0.02  max 0.70 | 0.25±0.20  min 0.03  max 0.55 | 0.47±0.54  min 0.03  max 1.86 | 0.31±0.37  min 0.02  max 1.86 |
| *sul1* [copies/ml] | 1.2×10^4^±1.5×10^4^  min 1.0×10^3^  max 5.3×10^4^ | 1.7×10^4^±1.5×10^4^  min 1.5×10^3^  max 5.1×10^4^ | 2.7×10^4^±2.7×10^4^  min 1.2×10^3^  max 8.9×10^4^ | 1.9×10^4^±2.0×10^4^  min 1.0×10^3^  max 8.9×10^4^ |
| *sul1* abundance^c^ [%] | 1.06±±1.61  min 0.08  max 5.73 | 1.42±1.82  min 0.04  max 6.48 | 1.58±1.51  min 0.03  max 5.0 | 1.36±1.62  min 0.03  max 6.48 |
| *sul2* [copies mL^-1^] | 4.3×10^2^±1.9×10^2^  min 1.2×10^2^  max 7.6×10^2^ | 6.3×10^2^±2.5×10^2^  min 1.7×10^2^  max 9.9×10^2^ | 7.2×10^2^±7.8×10^2^  min 1.5×10^2^  max 3.1×10^3^ | 6.0×10^2^±4.9×10^2^  min 1.2×10^2^  max 3.1×10^3^ |
| *sul2* abundance^c^ [%] | 0.053±0.084  min 0.001  max 0.292 | 0.051±0.055  min 0.002  max 0.181 | 0.083±0.151  min 0.004  max 0.544 | 0.062±0.102  min 0.001  max 0.544 |
| **Sediment** | | | | |
| Total heterotrophic bacteria [CFU g^-1^]^d^ | 1.9×10^5^±1.8×10^5^  min 2.7×10^4^  max 5.6×10^5^ | 5.0×10^5^±7.5×10^5^  min 2.8×10^4^  max 2.8×10^6^ | 1.1×10^6^±1.1×10^6^  min 5.8×10^4^  max 3.1×10^6^ | 5.8×10^5^±8.3×10^5^  min 2.7×10^4^  max 3.1×10^6^ |
| Coliforms [CFU g^-1^] | 8.6×10^3^±8.7×10^3^  min 6.2×10^2^  max 2.7×10^4^ | 1.0×10^4^±7.0×10^3^  min 9.0×10^2^  max 2.2×10^4^ | 4.9×10^4^±5.0×10^4^  min 6.7×10^3^  max 1.7×10^5^ | 2.3×10^4^±3.5×10^4^  min 6.2×10^2^  max 1.7×10^5^ |
| *intI1* frequency^b^ [%] | 1.6±2.2  min 0  max 7.3 | 1.3±1.4  min 0  max 4.2 | 3.9±5.4  min 1.0  max 16.7 | 2.3±3.5  min 0  max 16.7 |
| 16S rRNA gene [copies g^-1^] | 1.1×10^9^±1.4×10^9^  min 1.6×10^8^  max 5.1×10^9^ | 1.5×10^9^±1.4×10^9^  min 3.0×10^8^  max 3.8×10^9^ | 1.8×10^9^±1.4×10^9^  min 2.8×10^8^  max 5.4×10^9^ | 1.5×10^9^±1.4×10^9^  min 1.6×10^8^  max 5.4×10^9^ |
| *intI1* [copies g^-1^] | 5.5×10^5^±1.2×10^6^  min 7.9×10^4^  max 2.2×10^6^ | 1.2×10^6^±1.2×10^6^  min 1.7×10^5^  max 3.9×10^6^ | 1.4×10^6^±1.2×10^6^  min 2.3×10^5^  max 4.6×10^6^ | 1.0×10^6^±1.2×10^6^  min 7.9×10^4^  max 4.6×10^6^ |
| *intI1* abundance^c^ [%] | 0.46±0.75  min 0.02  max 2.78 | 0.38±0.39  min 0.05  max 1.42 | 0.49±0.44  min 0.07  max 1.49 | 0.44±0.54  min 0.02  max 2.78 |
| *sul1* [copies g^-1^] | 2.5×10^6^±4.0×10^6^  min 1.1×10^5^  max 1.4×10^7^ | 2.8×10^6^±4.2×10^6^  min 3.4×10^5^  max 1.5×10^7^ | 7.4×10^6^±9.4×10^6^  min 4.0×10^5^  max 3.1×10^7^ | 4.2×10^6^±6.6×10^6^  min 1.1×10^5^  max 3.1×10^7^ |
| *sul1* abundance^c^ [%] | 1.29±1.73  min 0.03  max 6.35 | 1.05±1.63  min 0.11  max 5.69 | 2.56±2.83  min 0.20  max 8.93 | 1.63±2.18  min 0.03  max 8.93 |
| *sul2* [copies g^-1^] | 3.5×10^3^±3.1×10^3^  min 0  max 6.9×10^3^ | 4.3×10^3^±4.6×10^3^  min 0  max 1.3×10^4^ | 7.1×10^3^±5.9×10^3^  min 0  max 1.6×10^4^ | 5.0×10^3^±4.8×10^3^  min 0  max 1.6×10^4^ |
| *sul2* abundance^c^ [%] | 0.00024±0.0003  min 0  max 0.001 | 0.00006±0.00009  min 0  max 0.0003 | 0.00012±0.00013  min 0  max 0.0004 | 0.00014±0.00020  min 0  max 0.001 |

^a^ Average ± SD.

^b^ Frequency of the *intI1* gene among culturable heterotrophic bacteria.

^c^ Abundance values expressed as percentages, calculated using the formula: [(gene copies/16S copies)×4×100], with four being the average number of copies of the gene encoding 16S rRNA per bacterial cell, according to the ribosomal RNA database [3, 21].

^d^ values per g of dry weight

**Supplementary Table S5** Mean values of bacterial counts, *intI1* gene among culturable bacteria, and copy number and abundance of 16S rRNA gene, *intI1*, *sul1* and *sul2* genes in water and sediment samples in different seasons

|  | **Winter** | **Spring** | **Summer** | **Autumn** |
| --- | --- | --- | --- | --- |
| **Water** | | | | |
| Total heterotrophic bacteria [CFU mL^-1^] | 1.2×10^4^ | 2.9×10^3^ | 6.7×10^3^ | 1.4×10^4^ |
| Coliforms [CFU mL^-1^] | 1.2×10^2^ | 2.1×10^2^ | 8.6×10^2^ | 2.4×10^2^ |
| *intI1* frequency^a^ [%] | 9.1 | 5.0 | 0.9 | 5.0 |
| 16S rRNA gene [copies mL^-1^] | 5.2×10^6^ | 6.2×10^6^ | 1.6×10^7^ | 1.1×10^7^ |
| *intI1* [copies/ mL^-1^] | 5.0×10^3^ | 1.9×10^3^ | 5.7×10^3^ | 3.6×10^3^ |
| *intI1* abundance^b^[%] | 0.65 | 0.17 | 0.26 | 0.17 |
| *sul1* [copies mL^-1^] | 9.6×10^3^ | 1.6×10^4^ | 2.5×10^4^ | 2.4×10^4^ |
| *sul1* abundance^b^ [%] | 1.19 | 0.96 | 1.54 | 1.73 |
| *sul2* [copies mL^-1^] | 4.6×10^2^ | 9.2×10^2^ | 5.2×10^2^ | 4.8×10^2^ |
| *sul2* abundance^b^ [%] | 0.062 | 0.141 | 0.041 | 0.005 |
| **Sediment** | | | | |
| Total heterotrophic bacteria [CFU g^-1^] | 3.2×10^5^ | 7.6×10^5^ | 3.8×10^5^ | 8.7×10^5^ |
| Coliforms [CFU g^-1^] | 1.3×10^4^ | 3.4×10^4^ | 2.7×10^4^ | 1.7×10^4^ |
| *intI1* frequency^a^ [%] | 1.5 | 3.5 | 0.6 | 3.6 |
| 16S rRNA gene [copies g^-1^] | 9.4×10^8^ | 2.7×10^9^ | 1.4×10^9^ | 1.2×10^9^ |
| *intI1* [copies g^-1^] | 5.7×10^5^ | 1.8×10^6^ | 1.3×10^6^ | 5.2×10^5^ |
| *intI1* abundance^b^ [%] | 0.47 | 0.54 | 0.49 | 0.27 |
| *sul1* [copies g^-1^] | 1.4×10^6^ | 6.1×10^6^ | 8.0×10^6^ | 1.3×10^6^ |
| *sul1* abundance^b^ [%] | 1.21 | 1.56 | 3.10 | 0.67 |
| *sul2* [copies g^-1^] | 6.1×10^3^ | 8.1×10^3^ | 6.1×10^3^ | 4.1×10^3^ |
| *sul2* abundance^b^ [%] | 0.00029^4^ | 0.00014 | 0.00003 | 0.00009 |

^a^ Frequency of the *intI1* gene among culturable heterotrophic bacteria.

^b^ Abundance values expressed as percentages, calculated using the formula: [(gene copies/16S copies)×4×100], with four being the average number of copies of the gene encoding 16S rRNA per bacterial cell, according to the ribosomal RNA database [3, 21].

**Supplementary Fig S1** Mean copy number and abundance of *intI1*, *sul1* and *sul2* genes in river water (A-B) and sediment (C-D) sampled in different seasons


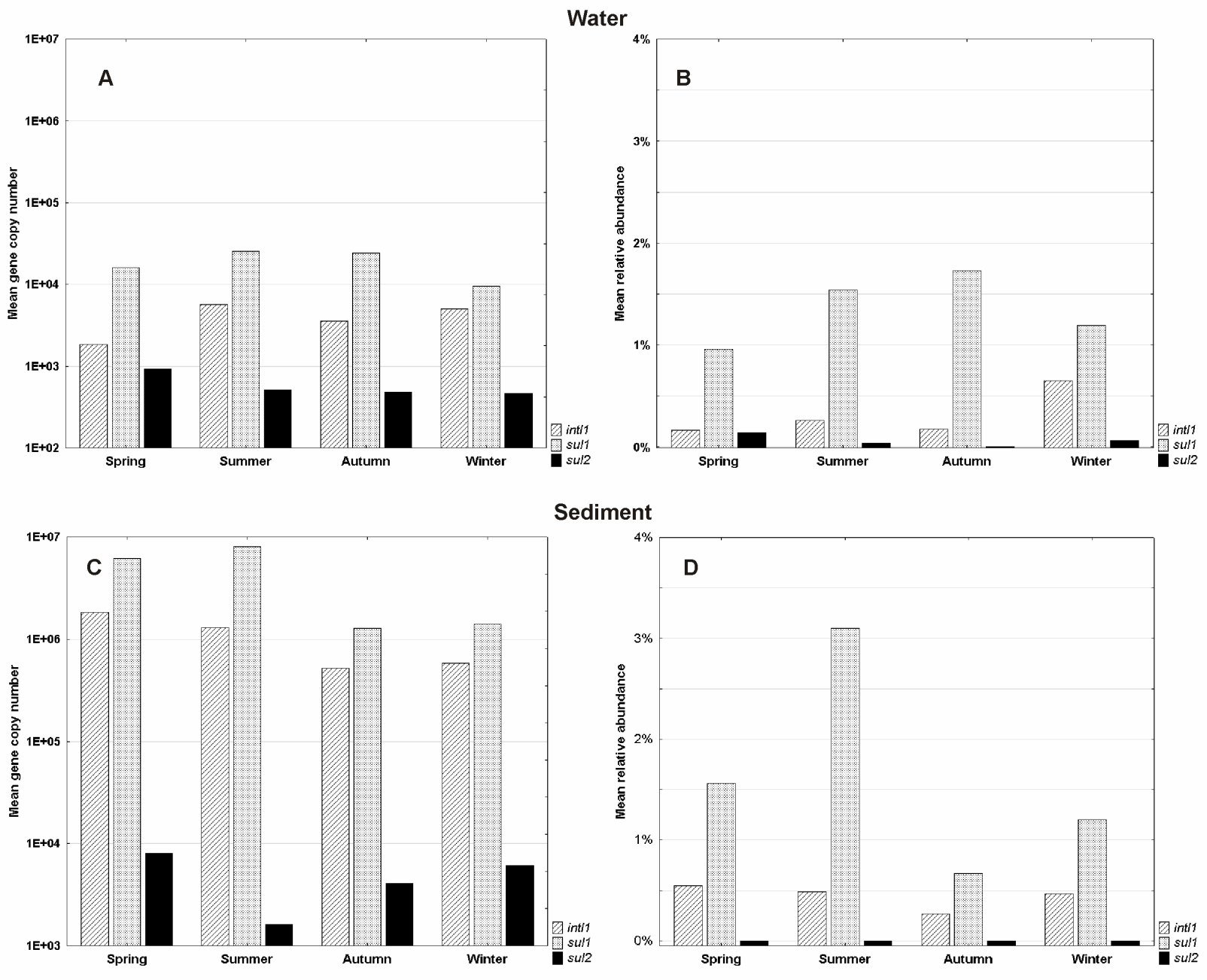

Supplement: Supplementary file 1 — (DOCX 577 kb) [file 248_2016_843_MOESM1_ESM.docx]
